# Implications of the Wilms’ Tumor Suppressor Wt1 in Cardiomyocyte Differentiation

**DOI:** 10.3390/ijms22094346

**Published:** 2021-04-21

**Authors:** Nicole Wagner, Marina Ninkov, Ana Vukolic, Günseli Cubukcuoglu Deniz, Minoo Rassoulzadegan, Jean-François Michiels, Kay-Dietrich Wagner

**Affiliations:** 1 CNRS, INSERM, iBV, Université Côte d’Azur, 06107 Nice, France; Marina.Ninkov@univ-cotedazur.fr (M.N.); ana.vukolic@roche.ch (A.V.); minoo.rassoulzadegan@unice.fr (M.R.); 2 Roche Glycart AG, 8952 Schlieren, Switzerland; 3 Stem Cell Institute, Ankara University, 06520 Ankara, Turkey; gunselicubukcu@gmail.com; 4 Department of Pathology, CHU Nice, 06107 Nice, France; michiels.jf@chu-nice.fr

**Keywords:** Wilms’ tumor suppressor 1, cardiomyocyte differentiation, mouse embryonic stem cells, myocardial infarction

## Abstract

The Wilms’ tumor suppressor Wt1 is involved in multiple developmental processes and adult tissue homeostasis. The first phenotypes recognized in Wt1 knockout mice were developmental cardiac and kidney defects. Wt1 expression in the heart has been described in epicardial, endothelial, smooth muscle cells, and fibroblasts. Expression of Wt1 in cardiomyocytes has been suggested but remained a controversial issue, as well as the role of Wt1 in cardiomyocyte development and regeneration after injury. We determined cardiac Wt1 expression during embryonic development, in the adult, and after cardiac injury by quantitative RT-PCR and immunohistochemistry. As in vitro model, phenotypic cardiomyocyte differentiation, i.e., the appearance of rhythmically beating clones from mouse embryonic stem cells (mESCs) and associated changes in gene expression were analyzed. We detected Wt1 in cardiomyocytes from embryonic day (E10.5), the first time point investigated, until adult age. Cardiac Wt1 mRNA levels decreased during embryonic development. In the adult, Wt1 was reactivated in cardiomyocytes 48 h and 3 weeks following myocardial infarction. Wt1 mRNA levels were increased in differentiating mESCs. Overexpression of Wt1(-KTS) and Wt1(+KTS) isoforms in ES cells reduced the fraction of phenotypically cardiomyocyte differentiated clones, which was preceded by a temporary increase in c-kit expression in Wt1(-KTS) transfected ES cell clones and induction of some cardiomyocyte markers. Taken together, Wt1 shows a dynamic expression pattern during cardiomyocyte differentiation and overexpression in ES cells reduces their phenotypical cardiomyocyte differentiation.

## 1. Introduction

The Wilms’ tumor 1 (Wt1) gene encodes a zinc finger protein that has multiple roles in embryonic development, adult health, and disease. Wt1 is an important regulator during embryogenesis [1,2,3,4] but is also involved in pathological processes, such as carcinogenesis. Originally proposed as a tumor suppressor, Wt1 is nowadays considered as an oncogene [5,6,7,8,9,10,11,12]. Wt1 is an evolutionary conserved transcription factor [13] with a high specificity for GC-rich regions [14]. Alternative RNA splicing results in formation of numerous protein isoforms, which can be classified into two major groups: Wt1(+KTS) and Wt1(-KTS), depending on the presence (+) or the absence (−) of three amino acids (lysine, threonine, and serine/KTS) in the linker sequence between zinc fingers 3 and 4 in exon 9 [15]. In general, it is thought that Wt1(-KTS) isoforms bind DNA with high affinity and regulate gene transcription [16], while Wt1(+KTS) isoforms have a higher affinity for RNA and might play a role in mRNA processing [17,18]. 

It is known that Wt1 regulates development and maintenance of various tissues of the cardiovascular, urogenital, nervous, hematopoietic, and immune system [19,20,21,22,23,24,25,26,27,28,29] through regulation of genes involved in proliferation, differentiation, and apoptosis—the essential processes for establishing early cellular fates within the embryo [23,24,30,31,32,33,34]. The crucial role of Wt1 in heart formation became clear when it was shown that homozygous deletion of Wt1 in mouse embryos was lethal, due to disturbed cardiac development [23,33]. Moreover, Wt1 cardiac conditional knockout mice died between E16.5 and E18.5 [35], when the heart should have achieved its definitive prenatal configuration [21,33,36]. Additionally, Wt1-deficient embryoid bodies failed to differentiate towards cardiac progenitor cells in vitro [35]. Wt1 is not only relevant for embryonic heart development but might also be involved in adult heart regeneration. Wt1 re-expression was noted in adult hearts following myocardial injury [37,38]. The role of Wt1 in adult cardiomyocytes is still controversial.

In development, the earliest recognizable structure in the growing heart is the primitive heart tube, which is formed at embryonic day 8.5 (E8.5), in the mouse [39]. Wt1 expression was first observed in a transitory cluster of cells—the proepicardium and the coelomic epithelium at E9.5. Wt1-positive proepicardial cells migrate across the pericardial cavity, proliferate, and spread over the surface of the myocardium to form the epicardial layer by E12.5 [40,41,42]. The highest proliferation levels and migratory capacity of epicardial cells correlate with elevated Wt1 expression during epicardial development [43]. Between E11.5 and E12.5, Wt1- expressing cells begin to migrate from the epicardium into the subepicardial zone to form a layer of subepicardial mesenchymal cells (SEMCs) [21]. Around E13.5, a subset of epicardial cells undergoes epithelial-to-mesenchymal transition (EMT), which induces the formation of epicardial-derived cells (EPDCs), a population of multipotent mesenchymal cardiac progenitor cells, which differentiate into the major cardiovascular cell types—cardiomyocytes, fibroblasts, smooth muscle, and endothelial cells [42,44]. The expression of Wt1 is essential for EMT and resulting differentiation of EPDCs and their derivates, through repression of the epithelial phenotype in epicardial cells [35,45]. However, additional consequences of Wt1 expression in cardiovascular progenitor cells are largely unknown. Generally, cardiovascular progenitors are defined by distinct combinations of cardiac-specific and stem cell associated genetic markers (Isl1, Nkx 2–5, c-kit, Oct3/4, Nanog). They maintain proliferative potential and are the main source of cardiomyocytes during development [46]. However, the impact of Wt1 expression on cardiomyocyte terminal differentiation was not studied in detail. Therefore, the purpose of this study was to examine how Wt1 affects the course of cardiomyocyte differentiation from progenitor cells during embryogenesis and adult life.

In the present study, we demonstrate Wt1 expression in cardiomyocytes during embryonic development, in the adult, and in response to injury *in vivo.* We show that transient Wt1 overexpression reduces phenotypic cardiomyocyte differentiation of ES cell clones *in vitro*, which is associated with modified expression levels of stem cell and cardiomyocyte marker genes.

## 2. Results

### 2.1. Wt1 Expression in Developing and Adult Hearts

**Figure 1:**
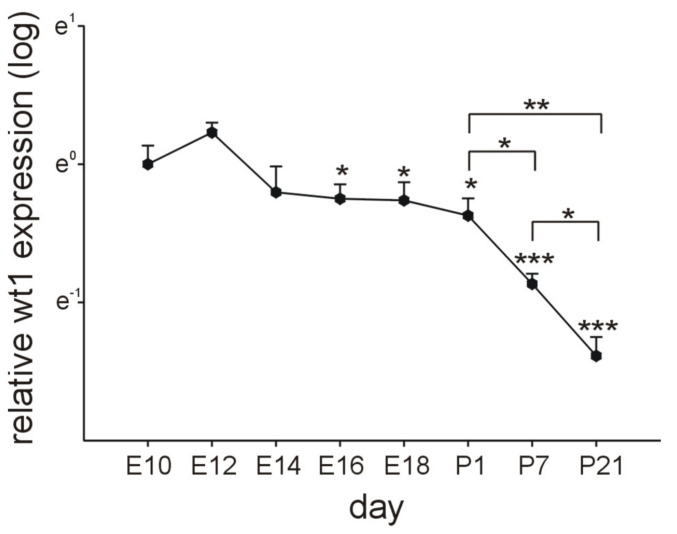
Cardiac Wt1 expression during embryonic development and after birth. Quantitative RT-PCRs for Wt1 in mouse hearts at different time points of embryonic development and after birth (*n* = 4 each, the four samples for E10 were each pooled from seven different organs, at E12 and E14 the four samples were pooled from four organs each). Expression of Wt1 was normalized to the mean of the respective Gapdh, actin, and Rplp0 expression. Next, the average of all samples at E10 was calculated. Individual samples were then normalized against this average value. Significance was tested between E10 and P21. Data are represented as means ± SEM. * *p* ˂ 0.05, ** *p* < 0.01, *** *p* ˂ 0.001.

To analyze systematically cardiac Wt1 expression during embryonic development and after birth, we measured its expression in heart tissue at different time points by quantitative RT-PCR (Figure 1). For this purpose, RNA was isolated from hearts including epicardium, myocardium, and endocardium, but excluding atria and outflow tract. In the developing heart of mouse embryos, we detected the highest Wt1 expression levels between E10.5 and E12.5, when the covering of myocardium with Wt1 positive progenitors should be accomplished [41]. In our study, Wt1 mRNA levels gradually decreased from E14.5, followed by a sharp drop after birth. Nevertheless, we were able to detect cardiac Wt1 mRNA expression until the end of observation period, at postnatal day (P) 21.

**Figure 2:**
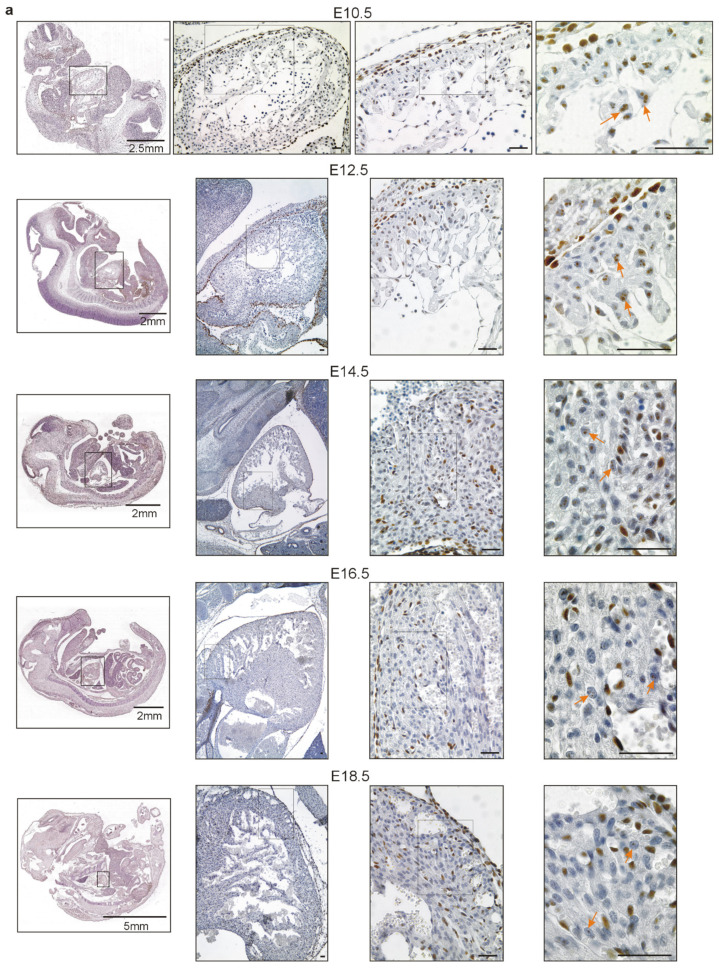

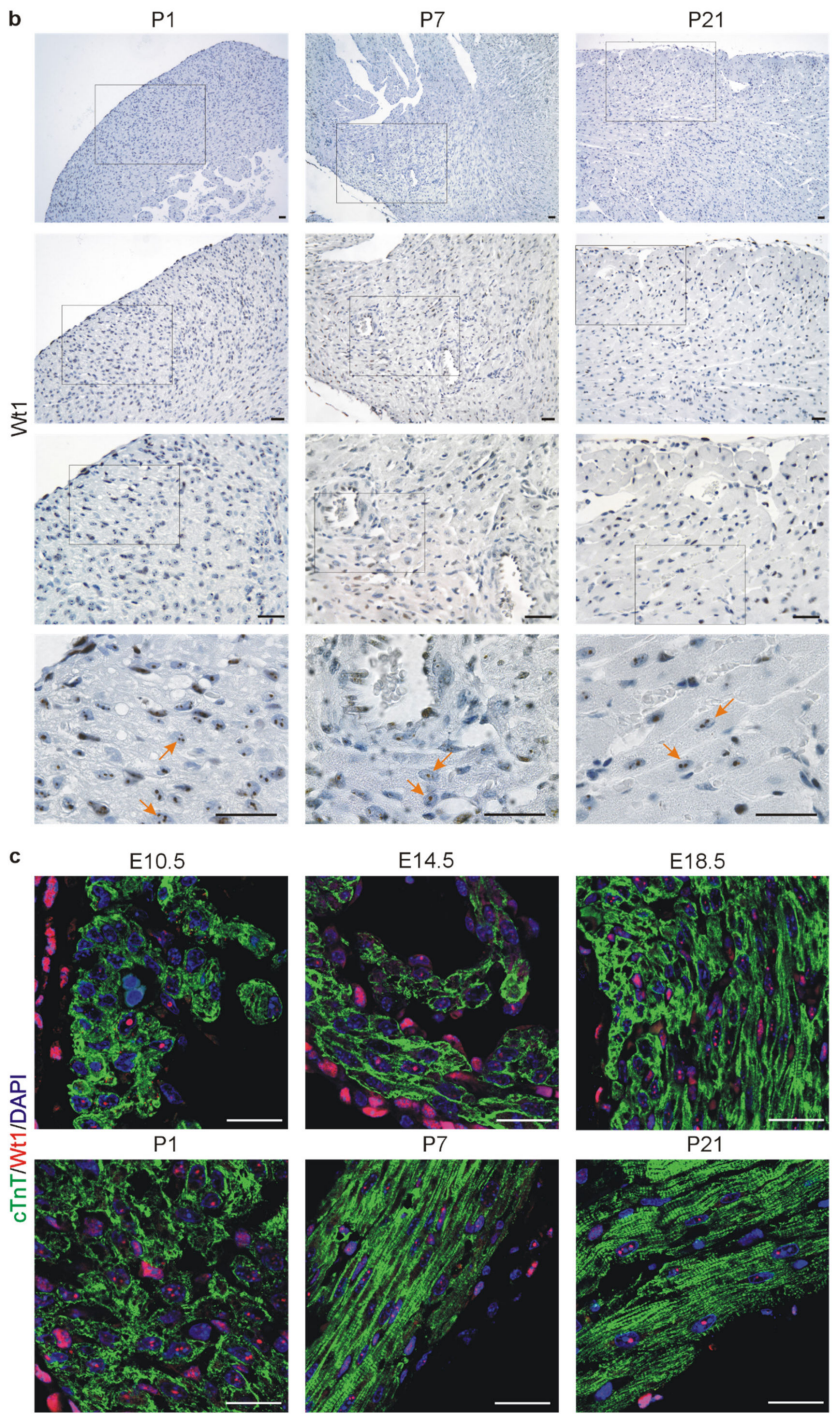
Wt1 is highly expressed in different cell types, including cardiomyocytes, during development of the mouse embryonic heart and persists in some cardiomyocytes after birth. Representative photomicrographs of Wt1 immunostaining on sections of mouse embryos (3,3′ diaminobenzidine (DAB) substrate, brown, hematoxylin counterstain) at different stages before birth (**a**) and of heart sections (**b**) after birth. Rectangles indicate the position of the magnification. Arrows mark Wt1 positive cardiomyocytes. (**c**) Confocal images of Wt1 (red)/cardiac troponin T (green) double-labeling on cardiac tissues at different time points of embryonic development and after birth. Nuclei were counterstained with DAPI (blue). Unless otherwise indicated, scale bars represent 50 µm.

Next, we performed immunohistochemical analyses of embryonic tissues and postnatal mouse hearts. Interestingly, we identified Wt1 positive cardiomyocytes in the heart from E10.5, the first time point analyzed, until adulthood (Figure 2). For Wt1 immunostaining of embryonic stages, we used paraffin sections of our mouse embryo collection. Rectangles in the scanned slides indicate the position of the higher magnifications in Figure 2. Only Wt1-positive cardiomyocytes are indicated by arrows as epicardial, endothelial, smooth muscle, and fibroblast expression of Wt1 had been reported already extensively [9,11,12,23,24,37,41,47,48,49,50,51,52,53,54,55,56]. At postnatal age, cardiac Wt1 expression diminished compared to the embryonic stages (Figure 1 and Figure 2), which corresponds to data reported in the literature [38]. However, in contrast to this report we show that Wt1 is not restricted to epicardium and endothelial cells, but it is still expressed in some cardiomyocytes after birth and in the adult (Figure 2). Interestingly, Wt1 expression in cardiomyocytes presents in a speckled manner, eventually suggesting a role of Wt1(+KTS). To confirm the histomorphologically observed expression of Wt1 in cardiomyocytes on a molecular level, we performed immunofluorescence double-labelling of Wt1 and cardiac troponin T, followed by confocal imaging for the different developmental stages (Figure 2c).

### 2.2. Wt1 Expression in Infarcted Hearts

**Figure 3:**
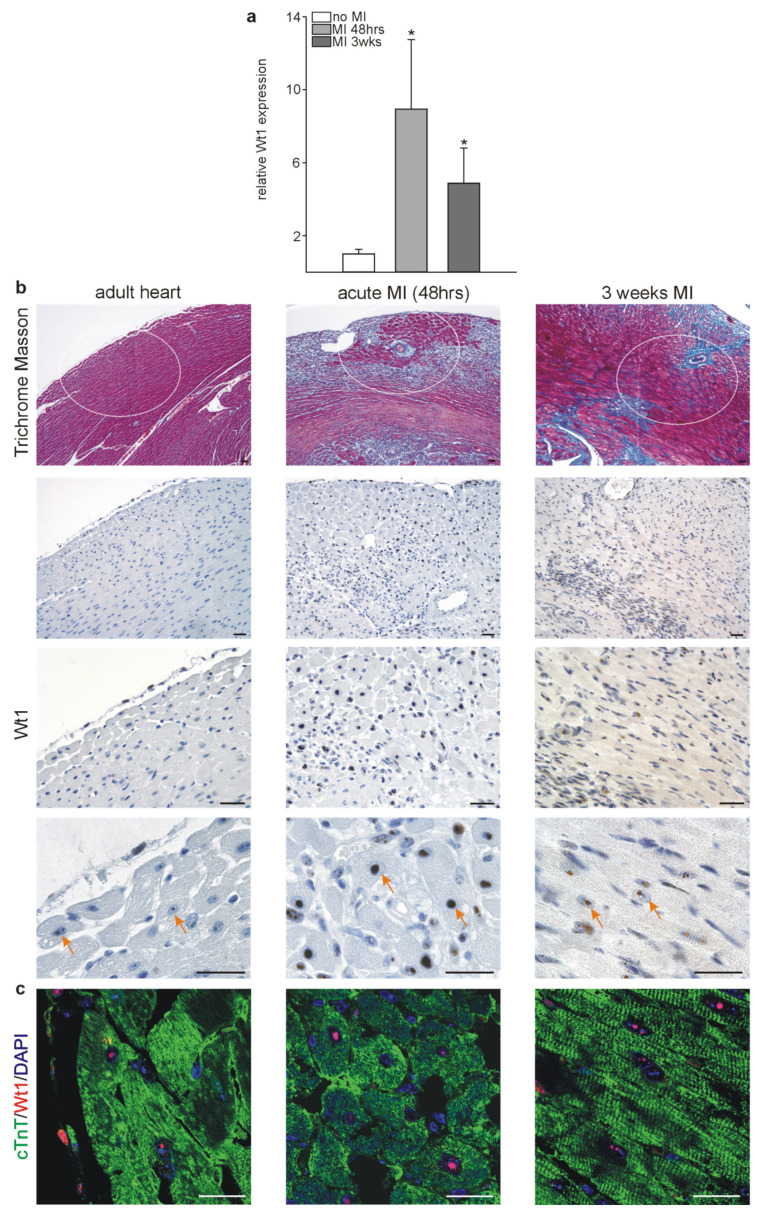
Wt1 is upregulated in cardiomyocytes after myocardial infarction. (**a**) Quantitative RT-PCRs for Wt1 in normal mouse hearts and hearts after 48 h or 3 weeks following LAD ligation (*n* = 4 for each group). (**b**) Upper panel: Trichrome Masson stainings for adult mouse heart and hearts 48 h or 3 weeks after myocardial infarction. Ellipses indicate the regions where subsequent photomicrographs of Wt1 immunostaining (panels below) for the adult mouse heart (3,3′ diaminobenzidine (DAB) substrate, brown, hematoxylin counterstain) were taken. Arrows mark Wt1 positive cardiomyocytes. (**c**) Confocal images of Wt1 (red)/cardiac troponin T (green) double-labelling of normal and infarcted mouse hearts after 48 h or 3 weeks after LAD ligation. Nuclei were counterstained with DAPI (blue). Data are presented as means ± SEM. * *p* ˂ 0.05. Scale bars represent 50 µm.

Next, we aimed at identifying the relevance of Wt1 expression under pathophysiological conditions in adult hearts. As similar processes are employed during organ development and regeneration, we hypothesized that Wt1 might contribute directly to cardiomyocyte cellular and functional differentiation in adult hearts following myocardial infarction (MI). Wt1 mRNA was determined by qRT-PCRs in hearts 48 h (acute phase) or 3 weeks (chronic phase) following left anterior descending coronary artery (LAD) ligation and in sham-operated controls without LAD ligation. Compared to control mice, a tenfold increase in Wt1 expression was measured 48 h following MI and a fivefold increase 3 weeks after ligation of the LAD (Figure 3a). Next, we employed immunohistochemistry to localize Wt1 expression in control and infarcted hearts of mice. In control hearts, only a few Wt1 positive cardiomyocytes were detected as described above, while the frequency was notably increased in acute MI samples especially in the border zone of the myocardial infarction, and more Wt1-positive cardiomyocytes, compared to controls, were still detected 3 weeks after MI (Figure 3b). The identity of a subset of Wt1 positive cells as cardiomyocytes was confirmed by colocalization of Wt1 (red) and cardiac troponin T (green) as cardiomyocytes markers within infarcted mouse hearts 48 h or 3 weeks after LAD ligation (Figure 3c). 

### 2.3. Cardiomyocyte Differentiation In Vitro

**Figure 4:**
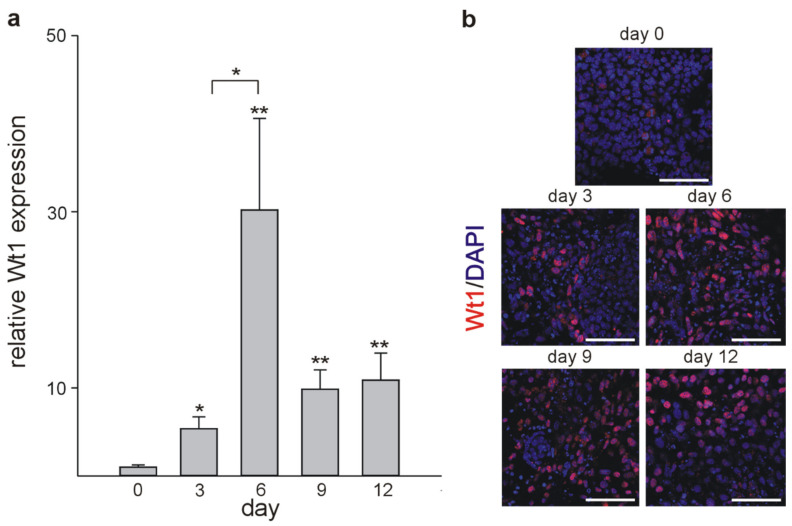
Wt1 is expressed in cardiomyocyte precursors and differentiated clones. (**a**) Quantitative RT-PCRs for Wt1 Randomly selected EBs were harvested on days: 0, 3, 6, 9, and 12 of culture. Expression of Wt1 was normalized to the mean of the respective Gapdh, actin, and Rplp0 expression. The Wt1 values are relative to Wt1 mRNA from day 0. The data from three independent experiments are represented as means ± S.E.M. * *p* ˂ 0.05, ** *p* < 0.01 vs. day 0. (**b**) Confocal images of Wt1 -labelling (red) of clones at the indicated time points. Cells were counterstained with DAPI (blue). Scale bars indicate 50 µm.

To get additional insights into the process of cardiomyocyte development, we used mouse embryonic stem cell (mESC) differentiation *in vitro*. mESCs have the potential to differentiate spontaneously into cardiomyocytes and represent a validated model for cardiac developmental investigations [57] as in vivo and in vitro cardiac cell differentiation employs the same signaling pathways [58]. Therefore, mESCs were differentiated as embryoid bodies (EBs) using the hanging drop method and their phenotype and temporal gene expression profiles were investigated. The first evidence of cardiomyocyte differentiation was the emergence of spontaneously beating clones from day 2 of EBs culture until day 21, when approximately 90% of clones exhibited rhythmic beating (Supplementary Figure S1). In line with our in vivo data, qRT-PCR analyses showed elevated Wt1 mRNA levels in ESC clones during cardiac differentiation. Wt1 mRNA levels increased until day 6 of culture. Afterwards average values decreased but remained above the basal levels measured on day 0 (Figure 4a). Immunocytochemistry for Wt1 in differentiating mESCs followed by confocal imaging confirmed, quantitatively, the results of the qRT-PCR analyses (Figure 4b).

As developmental changes are based on downregulation of embryonic genes and upregulation of those required for the adult differentiated phenotype, we analyzed temporal expression of stem cell (cKit, Sox2, Oct4, Nanog, Myc) (Supplementary. Figure S2) and cardiomyocyte markers (Nkx 2–5, Myh6, Myh7, Kdr, Pdgfra) (Supplementary. Figure S3) in randomly selected EBs on days: 0, 3, 6, 9, and 12 by qRT-PCR. As expected, the mRNA levels of the majority of stem cell markers started to decline from the earliest time point analyzed in differentiating mESC clones (Suppementary Figure S2). Expression of c-kit, however, did not diminish and followed the temporary expression pattern of Wt1 (Figure 4) without reaching statistical significance. Although the Myc gene has been described to be crucial for maintenance of pluripotency and self-renewal of mESC [59], expression of this stem cell factor was significantly downregulated only on day 9 of EBs culture (Supplementary Figure S2). Concerning cardiomyocyte markers, only Myh6 was significantly upregulated after 12 days of culture in randomly selected clones in this set of experiments (Supplementary Figure S3).

### 2.4. Wt1 Overexpression Affects the Course of Cardiomyocyte Differentiation

**Figure 5:**
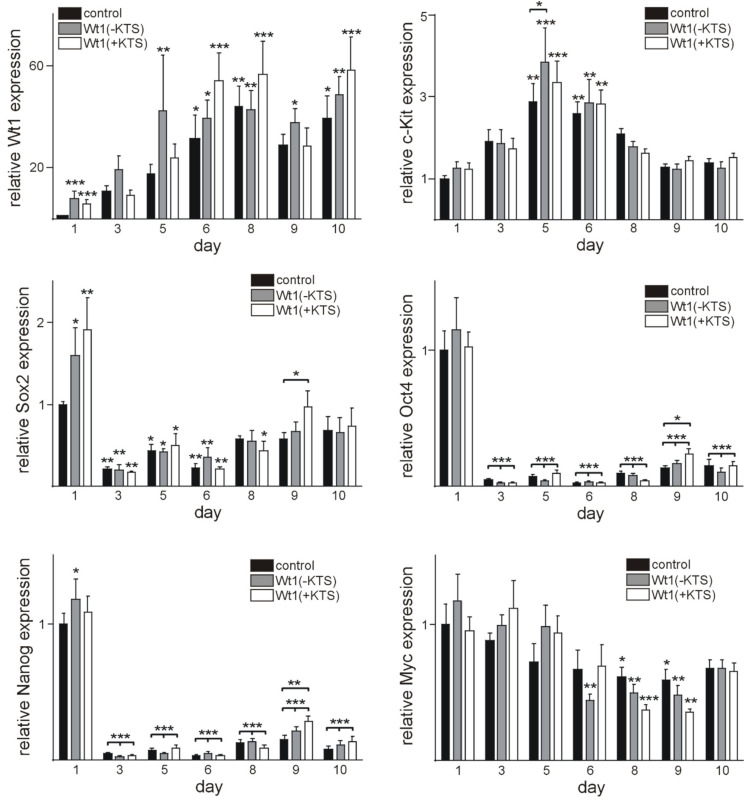
Time course of Wt1 and stem cell marker expression after electroporation of Wt1(-KTS), Wt1(+KTS), or empty vector control. Gene expression was quantified by qRT-PCR. Expression of each gene was normalized to the mean of the respective Gapdh, actin, and Rplp0 expression. The gene expression values are relative to respective control mRNA values (empty vector expression) at day 1. Data from four independent experiments including 12 independent randomly selected clones for each time point are represented as means ± S.E.M. and analyzed by Two-way ANOVA (Fisher’s LSD test). * *p* < 0.05, ** *p* < 0.01, *** *p* < 0.001.

**Figure 6:**
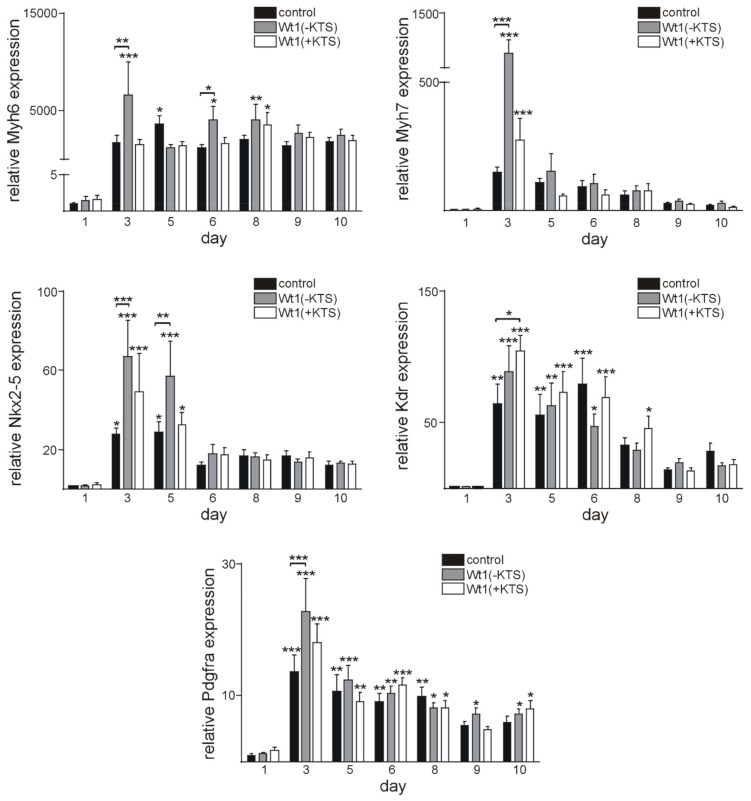
Time course of cardiomyocyte marker mRNA expression. Quantitative RT-PCRs for cardiomyocyte marker mRNA in control, Wt1(-KTS) and Wt1(+KTS) groups. Gene expression was normalized to the mean of the respective Gapdh, actin, and Rplp0 expression. Individual gene expression values are relative to 1 day post-transfection time point control mRNA values. The data from four independent experiments including 12 independent randomly selected clones for each time point are represented as means ± S.E.M. and analyzed by Two-way ANOVA (Fisher’s LSD test). * *p* < 0.5, ** *p* < 0.01, *** *p* < 0.001.

As Wt1 was highly expressed in embryonic hearts and differentiating EBs, we finally intended to define its impact on cardiomyocyte differentiation, on a cellular and molecular level. For this purpose, we transitionally transfected mESC with plasmids containing Wt1(-KTS), Wt1(+KTS), or empty vectors as control. The transfection efficacy was validated 1 day after electroporation. Wt1 expression was moderately increased in samples electroporated with Wt1(-KTS) and Wt1(+KTS) containing plasmids compared to controls at this time point (Figure 5). Regarding stem cell markers, Sox2 expression was higher in Wt1(-KTS) and Wt1(+KTS) transfected cells at this time point, while Nanog expression was significantly increased only in Wt1(-KTS) expressing mESCs. No significant differences in cardiomyocyte marker expression between Wt1 overexpressing cells and the respective controls could be detected at this early time point (Figure 6). 

Next, we analyzed the time course of Wt1 expression in empty vector control, Wt1(-KTS), and Wt1(+KTS) expressing clones (Figure 5). Wt1 expression increased on days 6, 8, and 10 of differentiation in the empty vector control group compared to the 1-day post-transfection time point. Significantly higher Wt1 mRNA values were observed on day 5, 6, 8, 9, and 10 compared to 24 h in the Wt1(-KTS) group, while significantly higher Wt1 mRNA levels were detected only on days 6, 8, and 10 in the Wt1(+KTS) group. The upregulation of Wt1 expression during cardiomyocyte differentiation in all groups resembles the situation in non-transfected cells, mentioned above, without an additional significant effect of the initial transient overexpression of the Wt1(-KTS) and Wt1(+KTS) constructs.

The time course of stem cell marker expression during cardiac differentiation of the transfected mESCs also resembled the expression pattern of non-transfected cells mentioned above. Beside the increase in Sox2 expression in Wt1(-KTS) and Wt1(+KTS) overexpressing cells and of Nanog in Wt1(-KTS) cells after 1 day, Oct4, Nanog, and Sox2 expression levels dropped rapidly in clones at day 3 of differentiation in all groups compared to day 1 controls. Surprisingly, Sox2, Oct4, and Nanog expression was higher in Wt1(+KTS) clones compared to empty vector control clones at the same time point. The decrease in Myc expression was less pronounced and reached statistical significance only at days 8 and 9 of differentiation in all transfected groups. Interestingly, c-kit expression showed a more dynamic pattern with significant increases on days 5 and 6 in Wt1(-KTS) and Wt1(+KTS) overexpressing clones and empty vector controls, when compared to the initial control values. A significant increase in c-kit in WT1(-KTS) clones on day 5 compared to empty vector transfected cells on the same time point is in agreement with our recent description of c-kit as a direct transcriptional target of Wt1 [9]. Afterwards, c-kit RNA levels returned to control values in all groups (Figure 5).

Next, we analyzed the effects of Wt1 overexpression on cardiomyocyte markers including Myh6, Myh7, Nkx2–5, Kdr, and Pdgfra. Kdr and Pdgfra represent factors, which are involved in cardiomyocyte differentiation, but not limited to cardiomyocyte progenitors [60,61]. Regarding the gene expression time course, qRT-PCR analyses showed the highest mRNA levels of all genes investigated on day 3 of culture, compared to 24 h for all three groups of clones. In addition, there were statistically significant increases in Nkx 2–5, Myh6, Myh7, and Pdgfra mRNAs in Wt1(-KTS) clones on day 3, compared to control clones at the same day, while in Wt1(+KTS) clones, only Kdr expression was significantly higher on day 3, compared to controls (Figure 6). 

**Figure 7:**
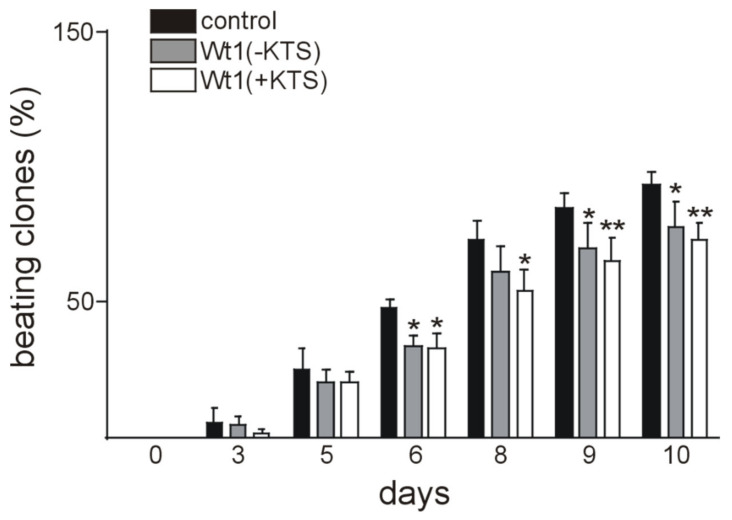
Wt1 overexpression delays functional cardiomyocyte differentiation. Wt1(-KTS), Wt1(+KTS), or empty vector controls were electroporated in mESCs. Hundred embryonic bodies were established per experiment and the number of beating clones counted at the indicated time points. Data from four independent experiments are represented as means ± S.E.M. * *p* < 0.05, ** *p* < 0.01.

Phenotype analysis of the embryoid bodies revealed that Wt1 overexpression (both Wt1(+KTS) and Wt1(-KTS) forms) resulted in lower rate of cardiomyocyte functional differentiation, compared to the controls (Figure 7, Supplementary Video S1). Beating clones were evident at day 2 in the control groups and at day 3 in Wt1(+KTS) and Wt1(-KTS) groups. Moreover, in the control group, the percentage of differentiated clones reached a plateau after day 10 and more than 90 % of EBs contained beating clones by the end of the observation period. At the same time, differentiation rate in Wt1(+KTS) and Wt1(-KTS) groups followed a slower dynamic with around 80 % of contractile clones by the end point of the experiments. The most pronounced drop in cardiomyocyte functional differentiation dynamics was found in the Wt1(+KTS) group, compared to controls. The percentage of phenotypically differentiated beating clones was significantly lower from day 6 until day 10 of the observation period in Wt1(-KTS) and Wt1(+KTS) clones (Figure 7). 

## 3. Discussion

The expansion of different cardiac cell types in a timely and spatiotemporal pattern is required for normal heart development (for review see [62]). It has been noted earlier that the Wilms’ tumor suppressor, Wt1, is required for murine heart development as Wt1 knockout mice have severely hypoplastic hearts and die during mid-gestation, most likely due to heart failure [21,33]. There is a general consensus that Wt1 expressing cells contribute to the development of endothelium, smooth muscle cells, and fibroblasts in the heart [12,23,24,35,37,38,47,48,62,63], but the contribution of Wt1-positive cells to cardiomyocytes during development and in cardiac repair still remains controversial. Earlier, a significant contribution of epicardial-derived cells to the cardiomyocyte lineage in the developing heart has been described based on lineage tracing experiments [42,44,56], which was questioned later [55,64,65]. Regarding a possible role of epicardial-derived Wt1 expressing progenitor cells for cardiac repair, the situation is comparable with some studies postulating an important role after myocardial infarction [52,66,67,68,69], while others did not confirm these results [70]. These contradictory results could be explained by different experimental approaches, staining procedures, limitations of the Wt1-Cre mouse models used [65], and by the fact that the re-activated epicardium is heterogenous and different from developmental epicardial cells [71] and only a fraction of cells in adult epicardium expresses Wt1 and is reliable targeted by the Wt1Cre lines [72]. For these reasons, we measured endogenous Wt1 mRNA levels and used a sensitive immunohistochemistry approach to characterize Wt1 expressing cells during cardiac development, in the adult, and during repair after myocardial infarction. The highest Wt1 mRNA expression was observed at E12.5. During this time window, Wt1 plays an important role in epithelial-mesenchymal transition (EMT) and mesenchymal epicardial-derived cell (EPDC) development through downregulation of E-cadherin, upregulation of Snail, and regulation of the Wnt/β-catenin signaling [35,53]. During later stages of embryonic and postnatal development, we observed a decrease in Wt1 mRNA expression, which is in agreement with previous results using a reporter system [21]. Nevertheless, some cardiomyocytes remained Wt1-positive even in the hearts of adult mice. It is conceivable that Wt1 regulates some of the cardiac progenitors by preventing terminal cardiomyocyte differentiation [73,74]. These progenitors could contribute to the cardiomyocyte lineage during development and give rise to the sparse de novo cardiomyocytes formation in adulthood [52]. 

Wt1 upregulation after myocardial infarction has already been reported several years ago [37]. However, earlier we focused mainly on the angiogenic response after myocardial infarction. It has been shown that Wt1 upregulation in adult heart vasculature was the response to local ischemia and hypoxia in rodents. It is thought that endothelial Wt1 expression is associated with neovascularization and recovery following MI [37,38]. The hypoxic conditions during development and after MI induced vascular formation via hypoxia-inducible factors, which directly upregulated Wt1 [49,75]. Wt1, in turn, regulates the expression of several angiogenic factors and receptors, positively [9,10,11,12,23,24,48,76,77,78]. De novo cardiomyocytes were reported to develop in adjacent areas of a myocardial infarction [79,80] and hypoxic regulation of Wt1 re-expression might also function to promote the tissue regeneration by cardiomyocytes differentiation from an activated progenitor pool [49,56,81]. Additionally, proinflammatory cytokines, such as TNF-α, IL-1β, and IL-6, could favor Wt1 activation after MI through activation of NF-κB. Furthermore, WT1 expression after injury might also be induced by soluble factors secreted by the myocardium [82]. This might induce progenitor cell proliferation and cell survival [83,84]. Given the distance to the epicardium, where we detected Wt1-positive cardiomyocytes already 48 h after myocardial infarction, it is unlikely that these cells are directly epicardium-derived. As the epicardium containing Wt1-positive cells promotes immune cell recruitment, neovascularization, and re-entry of cardiomyocytes into the cell cycle via mitogen secretion in response to injury (for review see [65,85]), both Wt1 expressing cell types might interact in repair. Interestingly, Tyser et al. [86] recently identified a common progenitor pool of the epicardium and myocardium by single cell transcriptomic analyses, most of the clusters expressing Wt1, which could explain expression in some cardiomyocytes and epicardium later in life. Developmental cardiac Wt1 expression diminished at the termination of heart formation, but we assume that low levels of Wt1 expression are sufficient to maintain a cardiac progenitor subset from terminal differentiation. This would support cardiac tissue regeneration by Wt1 reactivation when stimuli, such as hypoxia/inflammation, occur. However, more detailed examination of this pre-cardiomyocyte subset is necessary, as they could be employed as a valuable therapeutic tool for repair following myocardial infarction.

To obtain additional insights into the role of Wt1 in cardiomyocyte differentiation, we used cardiac differentiation of mESCs, a well-established model [57,58,87,88]. Wt1 was detectable in some undifferentiated mESCs and its expression levels increased along with cardiac differentiation until day 6. Wt1 expression in mESCs seems to be necessary for cardiomyocyte differentiation as *Wt1* null mESCs cells failed to differentiate towards the cardiomyocyte lineage [35]. To further characterize the role of Wt1 for cardiomyocyte differentiation of mESCs, we transiently overexpressed Wt1(-KTS) or Wt1(+KTS) constructs. Interestingly, this transient and moderate Wt1 overexpression reduced phenotypical cardiomyocyte differentiation, i.e., the percentage of beating clones throughout the observation period. At the onset of differentiation, signaling pathways regulating pluripotency of mESCs are inhibited through downregulation of stem cell genes, such as Sox2, Oct4, and Nanog [89]. This corresponds to our findings with significant downregulation of these genes at day 3 of differentiation when the first clones started beating. Whether the increase in Sox2 mRNA one day after Wt1(-KTS) and Wt1(+KTS) overexpression is directly related to activation by Wt1 remains to be clarified. Nevertheless, it might contribute to reduced phenotypic differentiation. The higher expression of Sox2, Oct4, and Nanog in Wt1(+KTS) transfected clones compared to control at day 9 reflects, most likely, an indirect effect of reduced differentiation. In contrast to these pluripotency factors, c-kit showed an increasing expression with a peak at day 5 of differentiation with a significantly higher value in Wt1(-KTS) transfected cells, compared to controls. This might be in agreement with c-kit representing a direct transcriptional target of Wt1 [9]. Increasing c-kit expression during mESCs differentiation is compatible with the induction of a cardiovascular progenitor phenotype [90]. Also increased expression of cardiac (Myh6, Myh7, Nkx2–5) and cardiovascular progenitor markers (Kdr, Pdgfra) [91] coincided with the onset of phenotypic differentiation of mESCs. Surprisingly, Myh6, Myh7, Nkx2–5, and Pdgfra were all expressed significantly higher in Wt1(-KTS) clones on day 3, while KDR mRNA was increased in Wt1(+KTS) overexpressing clones. Direct activation of Kdr by Wt1 has been documented already in murine developing gonads although this was mainly attributed to the Wt1(-KTS) isoform [76]. Whether the other differentially expressed genes identified here represent bona fide Wt1 target genes remains subject of further study. The temporary increases in Myh6, Myh7, Nkx2–5, and Pdgfra seem to not be sufficient to support long-term phenotypic cardiomyocyte differentiation, as clones with transient Wt1 overexpression showed less contractility. Whether this is related to short term increases in Sox2 and c-kit or other factors not identified in the present study remains an open question.

Although the mESC cardiac differentiation model is well established, the heterogeneity of clones, and cells within a clone, limit the use for further molecular and transcriptomic studies. The effects of transient Wt1 isoform overexpression and spontaneous increase in Wt1 expression upon cardiac differentiation of mESCs might, in addition, result in mixed outcomes. Regarding a potential role of Wt1 expressing progenitors for cardiac repair in vivo, a major limitation is currently the lack of techniques to isolate and expand these cells. 

## 4. Materials and Methods 

### 4.1. Mice and Tissue Preparation

All animal work was conducted according to National and international guidelines and was approved by the local ethics committee (Nice, France, 09.01.2013) (PEA-NCE/2013/106). 

Timed pregnant mice (NMRI) were purchased from Janvier Labs (Le Genest-Saint-Isle, France). Pregnant mice were sacrificed by cervical dislocation. Embryonic hearts were dissected, and tissues were used to prepare RNA. For immunohistochemistry, collections of paraffin-embedded whole embryos were used up to E18.5; for later stages, hearts were dissected. 

Myocardial infarctions were induced by ligation of the left coronary artery (LAD), as described elsewhere [92]. In brief, anaesthetized mice were endotracheally intubated, a thoracotomy between the third and fourth rib was performed, and the LAD was closed permanently with a 7–0 suture 2 mm distal to the left auricle. The thoracotomy and the skin wound were closed with 4–0 sutures and the mice remained intubated until spontaneous respiration was re-established. Animals were sacrificed at the indicated time points after infarction, the apex dissected for RNA preparation, and the remaining heart tissues used for paraffin-embedding followed by histological and immunohistological analyses.

### 4.2. Cell Culture

#### 4.2.1. Mouse Embryonic Stem Cell Culture

The mouse embryonic stem cell line, R1, was used. In order to stimulate proliferation and prevent differentiation, mESCs were cultured on a layer of mitotically inactivated primary mouse embryonic fibroblasts (MEFs)—feeder cells. The MEFs were prepared following the well-established protocol available online (http://www.ispybio.com/search/protocols/MEF_protocol.pdf, accessed on 4 January 2020). In short, a pregnant female mice was sacrificed by cervical dislocation around day 13.5 post coitum. Embryos were separated from the placenta. The head and dark red organs were removed, and the remaining tissue was minced with razor blades and then trypsinised until a single-cell suspension was obtained. Isolated MEFs were expanded in MEF medium (DMEM Glutamax/Gibco 61965-026 supplemented with 10% FBS/Gibco 10270-106; 1/100 l-glutamine/200 mM: Gibco 25030-024; 1/100 penicillin/streptomycin/10,000 U/mL, Gibco 15140-122, ThermoScientific, Cergy Pontoise, France) in 10 cm tissue culture dishes (Corning, NY, USA), until 90–100% confluence was reached. Then, cells were split at 1:4 ratio and MEFs from passages 3–5 at 80% of confluence were inactivated with mitomycin C (10 μg/mL, BML-GR311–0010, Enzo Life Sciences, Farmingdale, NY, USA) 3 h at 37 °C to generate feeder layers. Inactivated MEFs (iMEFs) were ready to use 24 h following mitomycin C treatment.

Mouse ESCs were grown on a feeder layer in an ESC medium composed of DMEM “Knock-out” (10829018) medium supplemented with 15% ES cell grade FBS (16141–002), 1/100 MEM non-essential amino acids (11140–035), 1/100 l-glutamine, 1/1000 2 mercapthoethanol (31350–010), 1/100 sodium pyruvate (11360–039), 1/100 penicillin/streptomycin—all from Thermo Scientific-Gibco. Prior to ESCs seeding, 10 µg/mL of mLIF was added to the medium (ESGRO Leukemia Inhibitory Factor supplement for mouse cell culture 10^7^ U/mL. Hemicon International, Inc., Temecula, CA, USA ESG 1107). After 48 h of culture, when mESCs formed multiple large colonies (100 % confluence), they were trypsinised and used further for differentiation or/and electroporation.

#### 4.2.2. mESC Differentiation by the Hanging Drop Method

The differentiation of mESCs was carried out, according to the protocol described by Wang and Yang [93]. Briefly, mESCs were resuspended in differentiation medium (ESC medium without mLIF supplementation). A tissue culture dish (10 cm) was filled with 10 mL of sterile PBS. Then, 50 drops of differentiation medium containing 500 cells/drop were placed to the lid of the dish and cultured for 72 h in order to form embryoid bodies (EBs). The drops with embryoid bodies (EBs) were then transferred to 96 well plates and incubated for the next 72 h. Following this incubation, EBs were transferred from 96 to 24 well plates coated with 0.2 % gelatin to enhance EBs attachment. The first day of EBs culture in 24 well plates was defined as day 0. The differentiation medium was changed daily and EBs were monitored for morphological and functional changes (contractility). At indicated time points, random samples were harvested for quantitative RT-PCR analysis.

### 4.3. Electroporation

For transient transfection of confluent undifferentiated mESCs cultured as mentioned in Section 4.2.1, plasmids containing either Wt1(−KTS) or Wt1(+KTS) expression vectors (Wt1 cDNA in pCB6+ plasmid), or empty vector as a control were used. For each group, 1 µg of plasmid was incubated with 3 × 10^6^ mESCs in 0.8 mL PBS 30 min on ice. Following the incubation, the electroporation was performed (400 V, 250 µF) using the Bio-Rad Gene pulser (Bio-Rad, Richmond, CA, USA). Once the electroporation was conducted, cells were quickly resuspended in ESC medium, plated on fresh feeder layers and incubated for 24 h to enable plasmid baseline expression prior to hanging drop culture, described in Section 4.2.2.

### 4.4. Quantitative RT-PCR 

Total RNA was extracted from mESCs, EBs, and organs using the Trizol reagent (Invitrogen, Carlsbad, CA, USA). The RNA pellet was dissolved in diethyl pyrocarbonate-treated H_2_O and RNA concentration was assessed spectrophotometrically. For reverse transcription, 0.5 µg of total RNA from mESCs and EBs was transcribed to cDNA using Maxima First Strand cDNA Synthesis kit (Thermo Scientific). The reverse transcription products were diluted 10× and 1 µl of diluted cDNA was used for quantitative PCRs. Detection of PCR products in real time was performed on the LightCycler Instrument (Roche Diagnostics, Mannheim, Germany) using the PowerUp SYBR Green Master Mix kit (Thermo Fisher Scientific, Waltham, MA, USA). For organs, first-strand cDNA synthesis was performed with 0.5 µg of total RNA using oligo(dT) primers and Superscript III reverse transcriptase (Invitrogen) (Table 1). One µl of the reaction product was taken for real time RT-PCR amplification (ABI Prism 7000, Applied Biosystems, Foster City, CA, USA) using a commercial SYBR^®^ Green kit (Eurogentec, Angers, France). Expression of each gene was normalized to the respective Gapdh, actin, and Rplp0 expression. For the in vivo part of the study, the average mRNA values of all samples at E10.5 were calculated; individual samples were then normalized against this average value. Data for mRNA from non-transfected cells were expressed as relative value to mRNA from day 0 (value normalized to 1); mRNA data for transfected cells were expressed as relative value to mRNA empty vector control 24 h post-transfection (value normalized to 1). 

### 4.5. Mouse Tissue Samples, Histology and Immunohistology

Samples from at least three different animals per time point were analyzed. Three µm paraffin sections were used for histological and immunohistological procedures. Haematoxylin-Eosin staining was routinely performed on all tissue samples. For Wt1 immunohistology, after heat-mediated antigen retrieval and quenching of endogenous peroxidase activity, the antigen was detected after antibody application (Wt1 rabbit monoclonal antibody, clone CAN-R9(IHC)-56-2, Abcam, Cambridge, UK,) using the EnVisionTM Peroxidase/DAB Detection System from Dako (Trappes, France). Sections were counterstained with Hematoxylin (Sigma, St. Louis, MO, USA). Omission of the first antibody served as a negative control. Additionally, some slides were incubated with an IgG Isotype Control (1:100, rabbit monoclonal, clone SP137, Abcam) as a negative control. Slides were photographed using a slide scanner (Leica Microsystems, Nanterre, France) or an epifluorescence microscope (DMLB, Leica, Germany) connected to a digital camera (Spot RT Slider, Diagnostic Instruments, Scotland). For immunofluorescence double-labelling of mouse hearts, anti-Wt1 rabbit monoclonal antibody from Abcam was combined with a mouse monoclonal anti-cardiac troponin antibody (clone 4C2, Abcam) using Dylight 594 donkey anti rabbit and Dylight 488 donkey anti mouse secondary antibodies (Jackson ImmunoResearch, Newmarket, Suffolk, UK). Cells were stained using anti-Wt1 rabbit monoclonal antibody from Abcam and Dylight 594 donkey anti rabbit secondary antibody. Negative controls were obtained by omission of first antibodies. Images were taken using a confocal ZEISS LSM Exciter microscope (Zeiss, Jena, Germany).

### 4.6. Statistical Analysis

Statistical analyses were performed using the GraphPad Prism software (version 6.2; GraphPad Software Inc, San Diego, CA, USA). Data are expressed as means ± standard error of the mean (S.E.M.). Statistical differences between mean values were assessed by analysis of variance (one-way or two-way ANOVA) followed by the Bonferroni post-hoc, Mann–Whitney, or Fisher’s test as indicated. A *p* value < 0.05 was considered to reflect statistical significance.

## 5. Conclusions

Here we show that Wt1 is expressed in cardiomyocytes during heart development and in adult life. Wt1 expressing cardiomyocytes might represent a subset of pre-differentiated cells that are able to contribute to regeneration of damaged heart tissue. On a cellular level, Wt1 kept a population of ESC derived cardiomyocytes from their final mature differentiated state through modulation of stem cell markers’ expression. A detailed analysis of the molecular mechanisms by which Wt1 regulates cardiomyocyte maturation will be subject of further studies. 

## Figures and Tables

**Table 1:** Primer Sequences.

Name	Sequence
**Wt1 forward**	CCA GCT CAG TGA AAT GGA CA [11]
**Wt1 reverse**	CTG TAC TGG GCA CCA CAG AG [11]
**Kit forward**	GCC TGA CGT GCA TTG ATC C [94]
**Kit reverse**	AGT GGC CTC GGC TTT TTC C [94]
**Sox2 forward**	CGC CCA GTA GAC TGC ACA
**Sox2 reverse**	CCC TCA CAT GTG CGA CAG
**Oct4 forward**	TGG GCG TTC TCT TTG GAA
**Oct4 reverse**	GTT GTC GGC TTC CTC CAC
**Nanog forward**	CAG GTT TCA GAA GCA GAA GTA CC
**Nanog reverse**	GGT TTT GAA ACC AGG TCT TAA CC
**Myh6 forward**	CCA AGA CTG TCC GGA ATG A
**Myh6 reverse**	TCC AAA GTG GAT CCT GAT GA
**Myh7 forward**	GCC TCC ATT GAT GAC TCT G
**Myh7 reverse**	CGC CTG TCA GCT TGT AAA TG
**Nkx2–5 forward**	ATT TTA CCC GGG AGC CTA CG
**Nkx2–5 reverse**	CAG CGC GCA CAG CTC TTT T
**Kdr forward**	AGT GGT ACT GGC AGC TAG AAG [94]
**Kdr reverse**	ACA AGC ATA CGG GCT TGT TT [94]
**Pdgfra forward**	ATG AGA GTG AGA TCG AAG GCA [94]
**Pdgfra reverse**	CGG CAA GGT ATG ATG GCA GAG [94]
**Rplp0 forward**	CAC TGG TCT AGG ACC CGA GAA G [95]
**Rplp0 reverse**	GGT GCC TCT GGA GAT TTT CG [95]
**Gapdh forward**	CCA ATG TGT CCG TCG TGG ATC T [48,95]
**Gapdh reverse**	GTT GAA GTC GCA GGA GAC AAC C [48,95]
**Actb forward**	CTT CCT CCC TGG AGA AGA GC [48,95]
**Actb reverse**	ATG CCA CAG GAT TCC ATA CC [48,95]

## Data Availability

Data is contained within the article or Supplementary Materials.

## Supplementary Materials

The following are available online at https://www.mdpi.com/article/10.3390/ijms22094346/s1.

## Abbreviations

DMEM	Dulbecco’s Modified Eagle Medium
E	Embryonic day
EBs	Embryoid bodies
EPDC	Epicardial-derived cells
FBS	Fetal bovine serum
LAD	Left anterior descending coronary artery
LIF	Leukemia inhibitory factor
MEF	Mouse embryonic fibroblasts
mESCs	Mouse embryonic stem cells
MI	Myocardial infarction
P	Postnatal day
pt	Post transfection
qRT-PCR	Quantitative reverse-transcription polymerase chain reaction
S.E.M.	Standard error of the mean
Wt1	Wilms’ tumor Suppressor 1
