# Supplementary material for: Implications of the Wilms’ Tumor Suppressor Wt1 in Cardiomyocyte Differentiation
Source: Int J Mol Sci. 2021 Apr 21;22(9):4346. doi: 10.3390/ijms22094346 (PMC8122684; doi:10.3390/ijms22094346)
Supplement: Supplementary file 1 [file ijms-22-04346-s001.zip › ijms-1190430-supplementary.pdf]

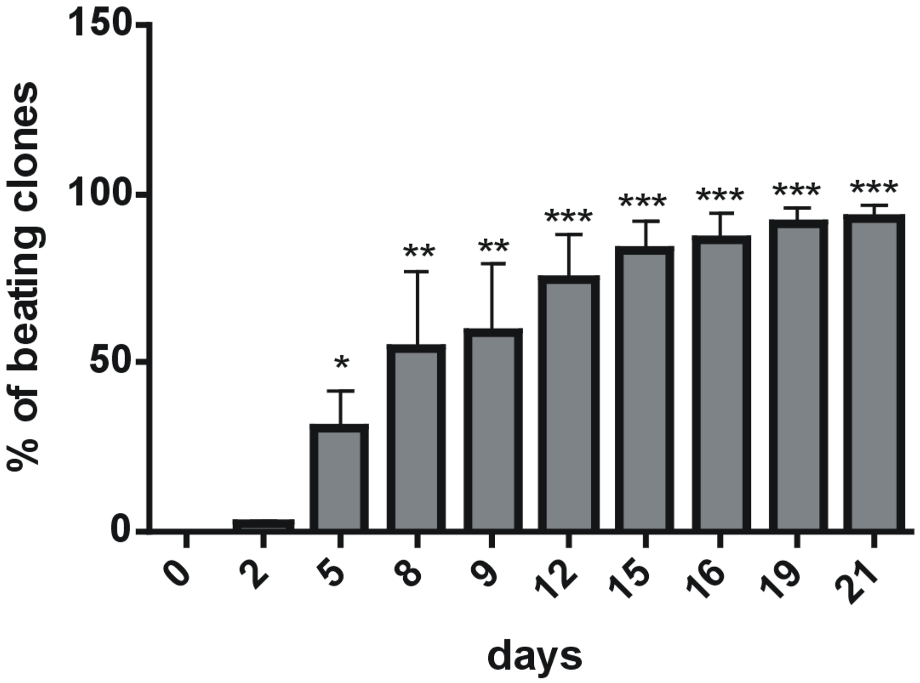

Supplementary Figure 1: Timeline of cardiomyocyte functional differentiation in embryonic body (EB) culture. Hundred EBs were established per experiment and the number of beating clones counted at the indicated time points. Data from three independent experiments are represented as means ± S.E.M. * indicates p<0.5, **p<0.01, and ***p<0.01 vs. day 0.

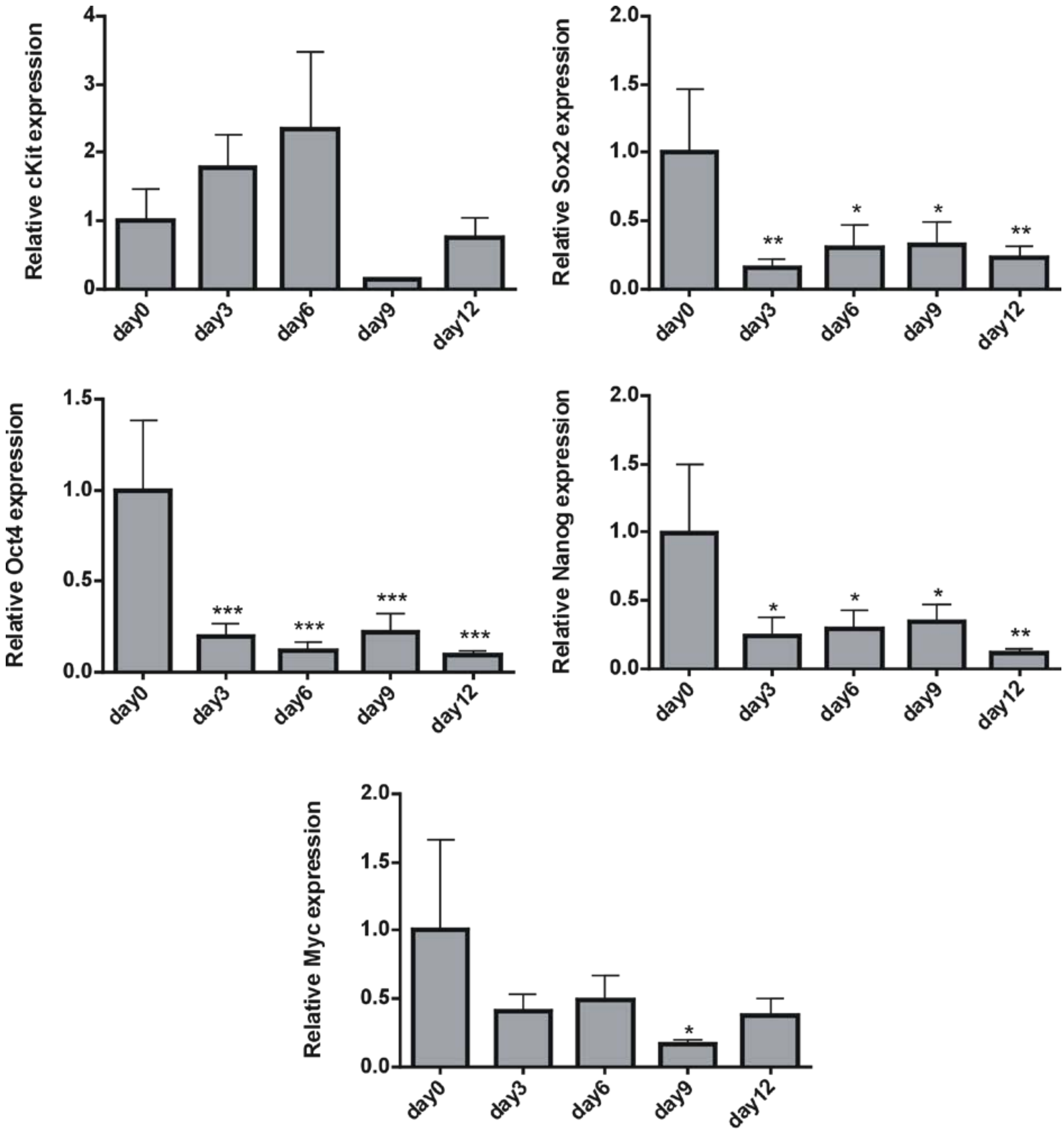

Supplementary Figure 2: Quantitative RT-PCRs for stem- and progenitor cell markers in ES cell clones. Randomly selected embryonic bodies were harvested on days 0, 3, 6, 9, and 12 of culture. Expression of each gene was normalized to the mean of the respective Gapdh, actin, and Rplp0 expression. The gene expression values are relative to respective mRNA expression from day 0. Data from three independent experiments are represented as means ± S.E.M. and analyzed by One-way ANOVA (Fisher's LSD test). *$p<0.05$, **$p<0.01$, ***$p<0.001$ vs. day 0.

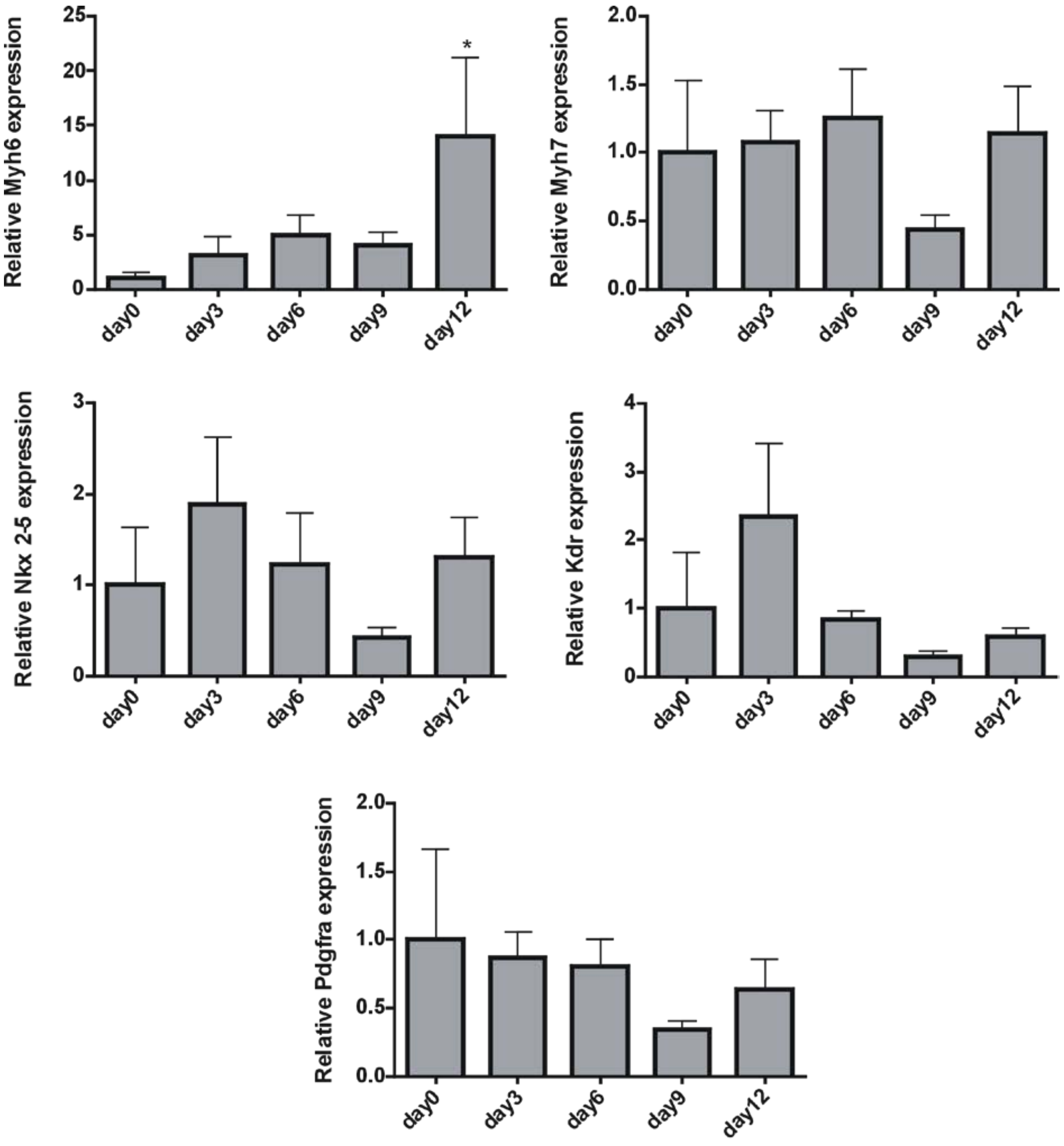

Supplementary Figure 3: Quantitative RT-PCRs for cardiac and cardiac progenitor cell markers in ES cell clones. Randomly selected embryonic bodies were harvested on days 0, 3, 6, 9, and 12 of culture. Expression of each gene was normalized to the mean of the respective Gapdh, actin, and Rplp0 expression. The gene expression values are relative to respective mRNA expression from day 0. Data from three independent experiments are represented as means ± S.E.M. and analyzed by One-way ANOVA (Fisher's LSD test). *$p<0.05$ vs. day 0.

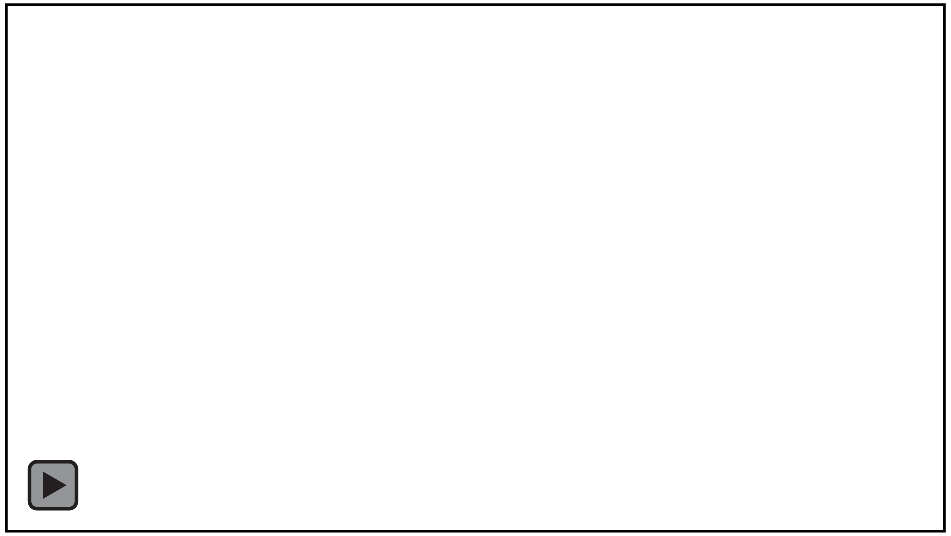

Control day 8

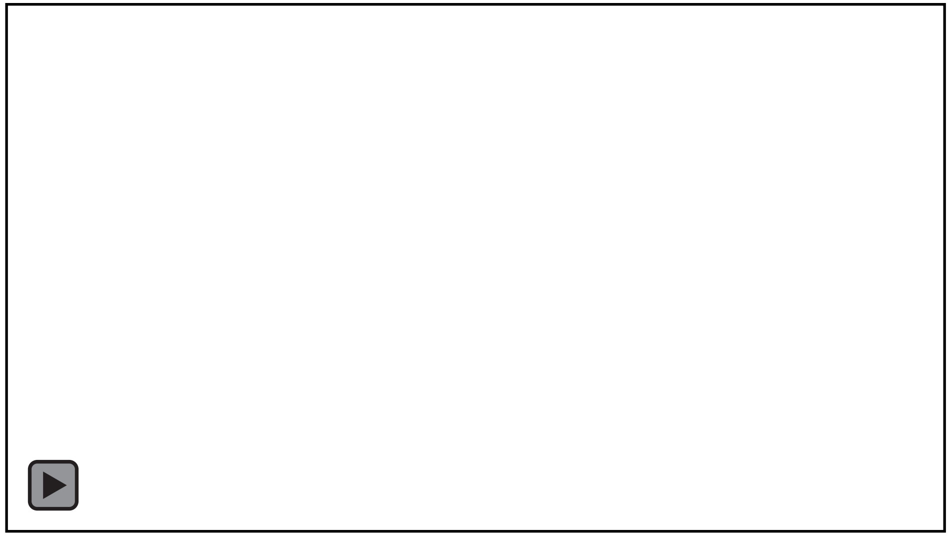

Wt1 (-KTS) day 8

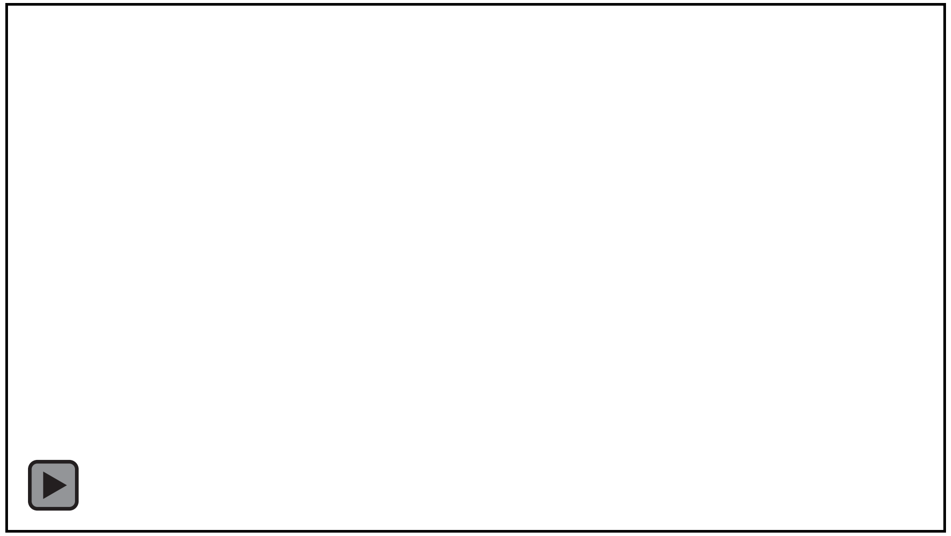

Wt1 (+KTS) day 8
